# Chloroplot: An Online Program for the Versatile Plotting of Organelle Genomes

**DOI:** 10.3389/fgene.2020.576124

**Published:** 2020-09-25

**Authors:** Shuyu Zheng, Peter Poczai, Jaakko Hyvönen, Jing Tang, Ali Amiryousefi

**Affiliations:** ^1^ Research Program in Systems Oncology, Faculty of Medicine, University of Helsinki, Helsinki, Finland; ^2^ Finnish Museum of Natural History (Botany), University of Helsinki, Helsinki, Finland; ^3^ Department of Biosciences, Viikki Plant Science Centre, University of Helsinki, Helsinki, Finland

**Keywords:** chloroplast genome, DNA barcoding, endosymbiosis, mitochondrial genome, photosynthesis, Plastomics, visualization

## Abstract

Understanding the complexity of genomic structures and their unique architecture is linked with the power of visualization tools used to represent these features. Such tools should be able to provide a realistic and scalable version of genomic content. Here, we present an online organelle plotting tool focused on chloroplasts, which were developed to visualize the exclusive structure of these genomes. The distinguished unique features of this program include its ability to represent the Single Short Copy (SSC) regions in reverse complement, which allows the depiction of the codon usage bias index for each gene, along with the possibility of the minor mismatches between inverted repeat (IR) regions and user-specified plotting layers. The versatile color schemes and diverse functionalities of the program are specifically designed to reflect the accurate scalable representation of the plastid genomes. We introduce a Shiny app website for easy use of the program; a more advanced application of the tool is possible by further development and modification of the downloadable source codes provided online. The software and its libraries are completely coded in R, available at https://irscope.shinyapps.io/chloroplot/.

## Introduction

Organelles play a pivotal role in eukaryotic cells by ensuring aerobic respiration and photosynthesis. Originating from independent endosymbiotic events – an important step in cellular complexity – organelles still maintain their own genomes (Ponce-Toledo et al., 2017; Martijn et al., 2018). Traditionally regarded as the powerhouses of cells, their core function is to produce the energy currency of the cell from foodstuffs (mitochondria) or from sunlight (chloroplast). However, they have key roles in other biological processes as well (Wolfsberg et al., 2001).

The genome of the chloroplast (a type of plastid) is typically 150–200 kb in size, and despite its conserved structure, some green plant lineages show reduction or expansion in size. The smallest plastid genome (~11 kb) was reported for the endoparasite *Pilostyles aethiopica* Welw (Apodanthaceae; Bellot and Renner, 2016), while the algal species *Haematococcus lacustris* (Girod-Chantrans) Rostafinski (Chlorophyceae) has the largest (1.35 Mb) known chloroplast genome (Bauman et al., 2018; Smith, 2018). Most chloroplast genomes have a quadripartite structure containing a large single-copy (LSC) and small single-copy (SSC) region and two identical copies of an inverted repeat (IRa and IRb) connected by junction sites (JS) in the genome. They encode ~120–130 genes, while only a small fraction of the genome is composed of non-coding intergenic regions. This implies a considerable evolutionary signal of the functional genes, as high purification pressure eliminates genetic content that is not of pivotal evolutionary importance (Wu et al., 2009). However, recent research suggests that genes of the chloroplast genome may not be as tightly linked as previously thought and, hence, may experience different evolutionary forces (Gonçalves et al., 2019). Mitochondrial genomes in metazoans (animals) are typically 15–20 kb in size and contain the same 37 genes used in energy production (Boore, 1999). In contrast, plant mitochondrial genomes are large and complex (191–11,319 kb) and are highly variable in size, arrangement, and repeat content, although coding sequences are highly conserved (Kozik et al., 2019). Both organelle genomes contain additional tRNAs, rRNAs, and other trans-encoded RNAs.

The first mitochondrial genome sequence was completed in 1981 for humans (Anderson et al., 1981), which was soon followed by the complete nucleotide sequences of tobacco (*Nicotiana tabacum* L.) and umbrella liverwort (*Marchantia polymorpha* L.) chloroplast genomes (Ohyama et al., 1986; Shinozaki et al., 1986). The major motivation of the first sequencing projects was to uncover the functional capabilities of organelles and to understand photosynthesis and energy production of plant and animal cells. After the conserved nature of genomes became apparent, PCR amplified fragments or barcodes of organelles became widely applied in investigating evolutionary relationships and population genetic changes. Universal primer sets are used to amplify popular barcodes in the chloroplast regions of plants (*mat*K, *ndh*F, or the non-coding *trn*T-F) and animals (COI; Waugh, 2007; CBOL Plant Working Group, 2009). Moreover, their smaller size and high copy number made them the most technically accessible regions of the genome, providing valuable information for comparative genome evolution, phylogenetic analysis, and population genetic studies (Ruhfel et al., 2014; Twyford and Ness, 2017; Amiryousefi et al., 2018b). Improvements in high-throughput sequencing technologies have made it possible to obtain complete organelle genome sequences from diverse organisms across the Tree of Life, at low costs. In addition, genome-skimming (Straub et al., 2012) has allowed short-reads to be obtained in a cost-effective way, which is suitable for the relatively small size of organelle genomes. Biodiversity projects have started to regard organelles as “super-barcodes,” since these sequences could still be obtained from degraded museum and herbarium samples (see Kistler et al., 2020). Thus, genome sequencing of plastids in plants and mitochondria in animals has become an essential tool in the study of evolution. This has led to a rapid increase in sequences deposited in public genomic databases. Currently, there are 5,180 chloroplast and 11,116 mitochondrial genomes deposited in NCBI Organelle Genome Resources (Wolfsberg et al., 2001), and their number continues to increase exponentially (see Tonti-Filippini et al., 2017). Many genome and transcriptome sequencing projects also generate large numbers of short-reads but discard organelle sequences as “contamination,” leaving an untapped wealth of resources for plastid and mitochondrial research.

It is also expected that large scale sequencing projects such as the Earth BioGenome (Lewin et al., 2018), 10 KP (Cheng et al., 2018), or the Darwin Tree of Life project^1^ will generate large amounts of data that will advance technological developments in “super-barcoding.” The current availability of data has already stimulated the development of different toolkits solely optimized for assembling (GetOrganelle: Jin et al., 2019; FastPlast: McKain and Wilson, 2017; MITObim: Hahn et al., 2013), annotating (Plann: Huang and Crong, 2015; MITOS: Bernt et al., 2013), and analyzing organelle genomes (Chlorobox: Tillich et al., 2017; CPGAVAS2: Shi et al., 2019). However, the analysis, interpretation, and visualization of biologically relevant results are still lacking further software development in key areas. For instance, despite their pivotal importance, only a small number of tools are dedicated to the graphical representation of physical genome maps. Currently, OrganellarGenomeDRAW (OGDRAW) is the most widely used program for the consistent and homogenous depiction of organelle genomes (Greiner et al., 2019) that are typically represented in a circular form. Besides this popular program, genome maps are also drawn manually or by other visualizing tools, e.g., GenomeVx (Conant and Wolfe, 2008) or CPGAVAS2 (Shi et al., 2019).

The growing number of deposited organelle genomes requires drawing tools with versatile coloring schemes capable of visualizing genome maps with customized colors. A drawing tool that is designed with a set of utilities and coloring schemes serves the basis for biological data representation. This ensures that the data representation makes biological sense and that aesthetically pleasing colors can be generated by accommodating sequential, diverging, and qualitative schemes easily understood by everyone, including those with color vision deficiencies. The comparison of genes and genomes also requires that unique structural elements such as the IRs of chloroplast genomes are correctly displayed. The assessment of these regions is the most challenging part of *de novo* plastid genome assembly with species showing expansion or contraction of the IRs. Plotting tools should be aware of errors and display possible incongruences arising from sequencing or assembly. With the recent advent of modern high-throughput sequencing methods, organelle visualization tools also need to display genomic variation detected among a set of individuals, populations, or various taxa. Plotting and characterizing nucleotide variation and allelic diversity across different species by plotting this information along the genome maps could help to select candidate genes in association studies or highlight “hotspots” for adaptive evolution or barcoding studies. Despite their useful capabilities, existing tools lack the mentioned characteristics in organelle genome visualization (see Table 1 and Supplementary Figure S1).

**Table 1:** Comparison of the major features of various software developed for the visualization of organelle genomes.

Feature	OGDraw v1.3.1	CPGAVAS2	GenomeVx	Chloroplot
Chloroplast	✓	✓	✓	✓
Mitochondria	✓	X	✓	✓
Circular visualization	✓	✓	✓	✓
Linear visualization	✓	X	X	X
Coloring schemes	X	X	✓	✓
Annotaton	X	✓	X	X
GC content	✓	✓	X	✓
Error aware IR detection	X	X	X	✓
Error highlighting	X	X	X	✓
Isomer representation	X	X	X	✓
Codon usage bias	X	X	X	✓
Costumized layers	X	X	X	✓
Restriction sites	✓	X	X	X
Transcript	✓	X	X	X
Repeat detection	X	✓	X	X

To provide an alternative visualization platform and to overcome the difficulties mentioned above, we developed a versatile tool for the graphical representation of organelle genomes. Our program not only signals the errors in specific genomic regions and presents the graphical map of genome structure but also allows the user to display different indices overlaid on genome maps. The online availability of our tool provides a flexible and user-friendly platform for the visual representation of organelle genomes.

## Materials and Methods

### Availability and Implementation

We are introducing Chloroplot, a comprehensive and interactive tool, for the visualization of organelle genomes. This online software is available at https://irscope.shinyapps.io/chloroplot/; it is completely coded in R, and its source code is also available for download for modified use and further development. The program was tested on more than 100 carefully selected sequences and also deposited online, including all major groups of eukaryotes (mitochondrial genomes). For chloroplasts, we sampled all major groups of the plant kingdom (Archaeplastida) to test and construct the optimal performance of the software (Supplementary Table S1). The visualization is optimized for genomes 16–400 kb in size, which fits most mitochondrial and chloroplast genomes targeted by sequencing efforts. Larger genomes (>400 kb) can also be drawn with Chloroplot after downloading and running the freely available R code.

### Data Input and Usage

**Figure 1:**
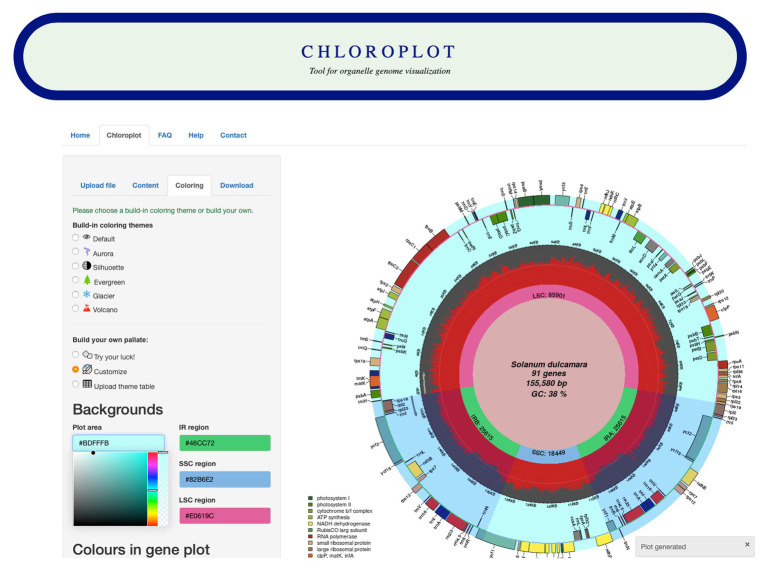
Screenshot of the Chloroplot online web interface. Chloroplot gives vast control over the generated output with respect to the predefined color schemes and interesting genomic indices.

The input data for Chloroplot are either chloroplast or mitochondrial genome annotations. These files should be in standard GenBank file format (“.gb suffixed files”), and the user has the option to either feed in the NCBI GenBank accession number of the taxa or alternatively, upload a compatible GenBank file. The structure of the data should be strictly preserved, as the downstream analysis is based on the data format embedded in the GB file. Once the input data are uploaded, users can choose different configurations for plotting different information (Figure 1). These options provide several choices for scientific indices and aesthetic layers, from codon usage bias for each gene to different color schemes for a unique representative output. The users also have an option to customize the coloring of the plot according to their preferences and to save their color scheme for later use.

## Results

**Figure 2:**
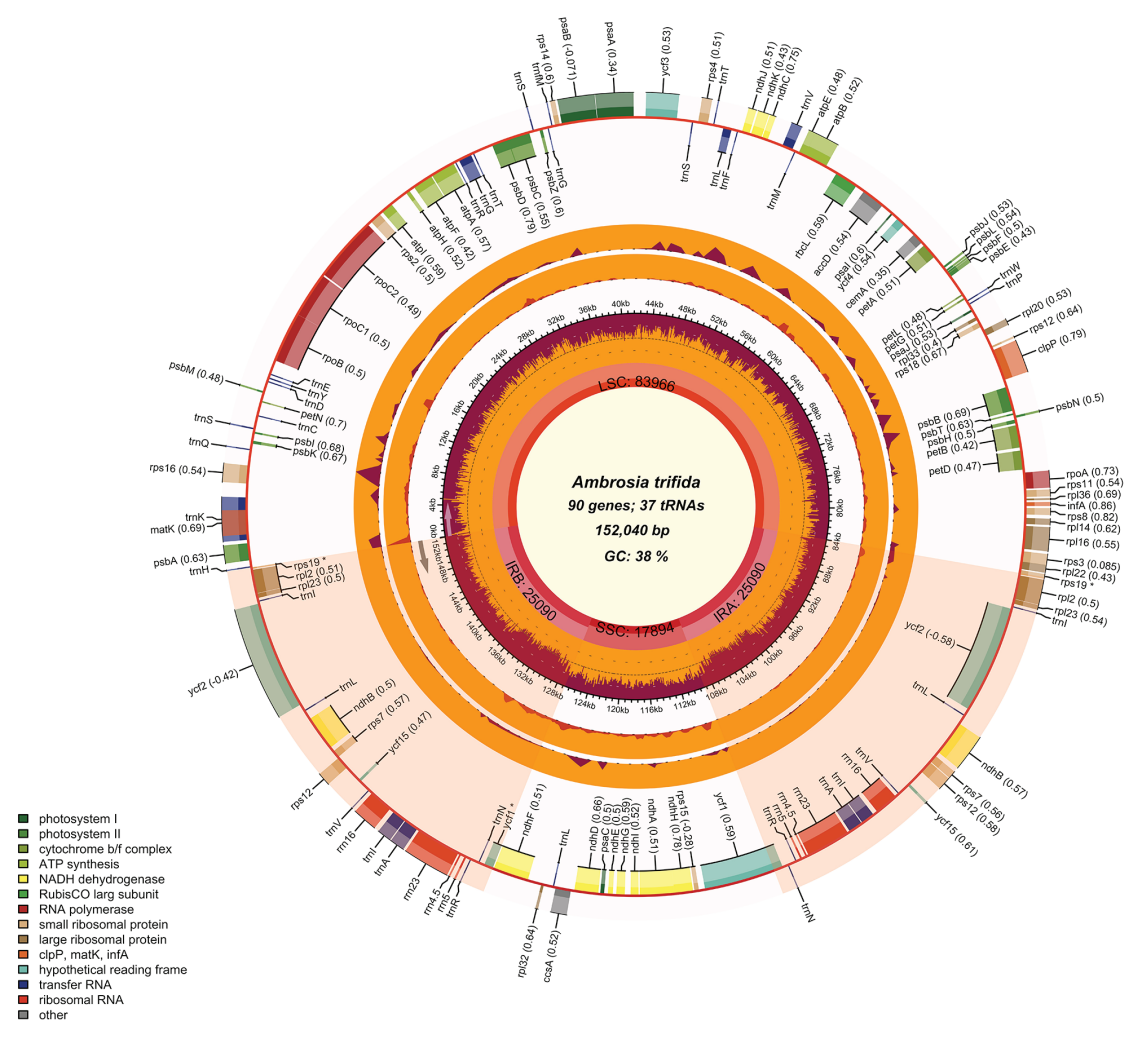
Chloroplast genome map of giant ragweed (*Ambrosia trifida* L.). The species name and specific information regarding the genome (length, GC content, and the number of genes) are depicted in the center of the plot. In the first inner circle, the optional GC content is depicted as the proportion of the shaded parts of each section. The length of the corresponding single short copy (SSC), inverted repeat (IRa and IRb), and large single-copy (LSC) regions is also given. The gradient GC content of the genome is plotted in the second circle with zero level based on the outer circle. Extending outward, the next two layers are the optional input indices of the nucleotide and indel diversity of *A. trifida* compared with *Ambrosia artemisiifolia*, respectively. The gene names and their optional codon usage bias are labeled on the outermost layer, with pseudogenes marked with asterisks. Genes are color-coded by their functional classification. Represented with arrows, the transcription directions for the inner and outer genes are listed clockwise and anticlockwise, respectively. The optional gene specific GC content is depicted with the proportion of shaded areas. The optional shaded area stretching from the inner sphere toward the outer circle marks the IR regions.

By automatically distinguishing the type of organelle genome as being either mitochondrion or chloroplast, Chloroplot generates a scaled circular plot as a schematic representation of the input genomes. Chloroplot also allows vast control of the different configurations related to the calculative indices, as well as the appearances of the result. Finally, the output graph is available for download in various selective formats in a wide range of resolutions (Figure 2). The major functions of Chloroplot are listed below.

### Precise Representation of the Fundamental Genomic Structure

**Figure 3:**
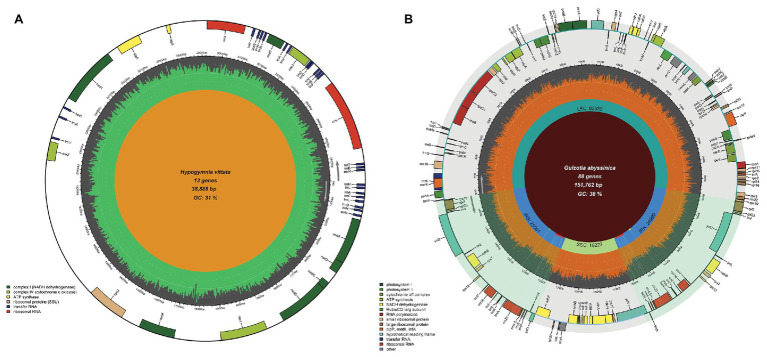
Mitochondrial genome map and chloroplast IR mismatching capabilities. Chloroplot can automatically distinguish organelle genome types and plot the respective elements of each genome, as well as detect the differences between IR regions in the plastid genome. **(A)** The mitochondrial genome map of *Hypogymnia vittata* (Ach.) Parrique (1898) with configurable functional gene families. **(B)** The detection of SNPs, insertion, and deletion of the nucleotides causing differences in the two IR regions of *Guizotia abyssinica*. The bold dots on the inner circle of the IR regions, each with a corresponding shadow extending outward with green, red, and yellow colors indicate the existence of the insertion, SNPs, and deletion on the corresponding genomic coordinates, respectively.

In the case of chloroplast genomes, Chloroplot has many special features that are designed to represent its dynamic nature and depict its distinct quadripartite structure. This organization of plastid genomes is formed by two identical reverse complement stretches of the nucleotide sequences called IRs, which separate the large single-copy (LSC) and small single-copy (SSC) regions. Thus, the IRs form four connecting points within the genome called JS. These sites often bear signs of evolutionary sweeps and drifts among lineages, making their depiction and identification crucial for comparative studies (Amiryousefi et al., 2018a). In addition to the overall guanine-cytosine (GC) content and the classification and representation of functional genes on the plot, Chloroplot is specifically designed to detect these unique structures of plastid genomes and represent their minute positions on the plot. In the case of the mitochondrion input where this quadripartite structure is missing, the program automatically activates the pre-assigned function for only plotting the GC content and the corresponding functional gene families related to the mitochondrion (Figure 3A).

### Inversion of Specific Regions

Chloroplot depicts the base composition embedded in the annotation file and enables visualization of the flip-flopping orientation of the SSC in chloroplast genomes occurring in an equimolar ratio in a single plant cell (Wang and Lanfear, 2019). Chloroplast DNA within an individual exhibits a form of heteroplasmy defined by the inversion isomers of the SSC that differ in their relative orientation (Palmer, 1983). This is an important fact, as the bulk of downstream analyses and comparative conclusions are unwittingly based on the false impression that this portion of the genome is unique and stable. In fact, this has led to a flaw in some studies that have misinterpreted the inversion isomers as mutational hotspots in diverse plant lineages (see Walker et al., 2015). Currently, chloroplast genomes can be deposited in NCBI without any preferable isomer orientation, leading to an apparent variation in the SSC. Chloroplast genomes are often assembled using a reference guided protocol propagating the orientation of the isomer. A distinct feature of Chloroplot is its ability to reverse complement the SSC regions embedded in the configuration panel. The possibility of swapping between the two inversion isomers of the SSC can help to avoid laborious manual editing, as well as overlooking this natural phenomenon. For more efficient representation of the genome, Chloroplot also allows the reverse complementing of the LSC and IR regions. In cases, where a gene is extending beyond a region – and hence, reverse complementing this region would bisect the gene – Chloroplot automatically scans the other side of the region when deciding and labeling the corresponding genes as pseudo or functional.

### Presentation of Non-identical IRs

The presence of IRs is nearly a universal feature of the chloroplast genomes of land plants. There are several indications that IRs are subjected to operating gene conversion and copy correction mechanisms (Goulding et al., 1996). Since concerted evolution homogenizes the nucleotide composition of the IRs among abundant copies in plant cells, their sequences should be identical. Non-identical IR copies frequently arising from poor genome assembly, read processing, and quality assessment are often undetected, which can lead to erroneous interpretations. For example, the plastid genome sequence of *Guizotia abyssinica* (L.f.) Cass (Dempewolf et al., 2010) contains non-identical IRs, possibly arising from sequencing errors, which has accidentally slipped the authors attention. The default settings for automatic IR detection embedded in OGDRaw assume that IR sequences are completely identical (Lohse et al., 2013); otherwise, the algorithm terminates the search when single nucleotide polymorphisms (SNPs) are encountered in the sequences. Considering that sequence conversion is acting over the IRs, this is the proper methodological procedure, since OGDraw also allows the visualization of non-identical IR sequences using the manual function. This assumes that users carry out quality controls before graphically displaying their chloroplast genome maps. The example of *G. abyssinica* shows that errors might slip the attention of researchers, and an error-aware IR detection method would help reduce such error rates. In the case of *G. abyssinica*, the junction sites JSB (IRb/SSC) and JSA (SSC/IRa) are located in the rrn23 gene of the 23S rRNA, indicated by Dempewolf et al. (2010). On the other hand, the IRb stretches 3,235 bp further from the SNP hotspot in the *ycf*1 gene, which is duplicated as a truncated pseudogene in the IRa. The junctions shown for *G. abyssinica* (Dempewolf et al., 2010) are locations for three SNPs in the rrn23 gene where OGDraw terminated the IR search. When working with chloroplast genome data, Chloroplot can automatically detect IR regions even if their sequences are non-identical and when short stretches of SNPs, insertions, and/or deletions are present in the input files. The IR regions are detected algorithmically in the following steps: (1) Genome extraction: Chloroplot first extracts the genome sequence from the GB file; (2) Sub-genome sequence formation: depending on the length of the extracted genome sequence, Chloroplot sets an adaptive window length which slides base by base along the genome sequence to produce sequence stretches; (3) Mapping to the reverse complement genome sequence: Chloroplot then maps the adaptive sized stretches from step 2 to the reverse complement genome sequence. The mapping results indicate the start and end position for IR regions; (4) Adjusting start and end points: as it is possible that the origin of the genome is inside IR regions, Chloroplot double checks whether the coordinates from step 3 include the first or last base pair of the genome. In such a case, Chloroplot shifts the origin point forward or backward with the length of the window size and repeats the process of step 2–4 until it converges; and (5) Detecting mismatches in IR regions: two IR sequences are extracted from the genome sequence according to the start and end positions acquired above. Pairwise alignment is used to detect the mismatches (SNP, insertion, and deletion) in two IR sequences. The formulation of the IR detection engine of Chloroplot allows differences between the IRs to be highlighted if their sequences are non-identical. If insertions, SNPs, and deletions are present among repeats, they will be marked with green, red, and yellow dots, respectively, on the IR regions, and extending white shadow rays mark the position of the differences (Figure 3B).

### Gene-Specific Indices and Overlaid Parameters

Despite the lack of sexual recombination, recent evidences showed that organellar genomes undergo intra- and inter-molecular recombination, which generate divergence among copies (Oldenburg and Bendich, 2016; Kobayashi et al., 2017; Ruhlman et al., 2017). Thus, some contemporary studies are aiming to uncover the mechanisms of organelle genome mutation, selection, and inheritance. Other studies aim to demonstrate the capabilities of these genomes in species barcoding and population genomic investigations. Such “super-barcoding” studies are interested in identifying mutational hotspots that can be used in further marker development. Both contemporary research directions require the depiction of additional genomic information plotted together with the scaled genome maps. Means of nucleotide diversity (*π*) defined as nucleotide differences per site between two randomly chosen sequences or the average number of nucleotide differences (*k*) are good examples that can provide helpful information for these studies. Besides fine-scaled drawing, our software is also capable of plotting three user-defined extra layers of information on the genome map. In these cases, the information needs to be uploaded separately in a predefined format. This could include any measures related to recombination or diversity represented on a numerical scale accompanied by genomic coordinates (Figure 2). In addition to this basic representation of genes, the interactive configuration of the program allows plotting the diverse information about the genome such as codon usage bias and IR coordinates.

## Conclusion

Chloroplot is a generic online visualization tool specifically designed to reflect the genetic architecture of organelle genomes. Furthermore, with the obvious errors in these genomes that arise from sequencing and assembly, Chloroplot can both indicate and bypass such shortcomings, as well as detect the correct position of the IR regions and represent the flip-flopping of the SSC. While the quality of the input file is still of immense importance, Chloroplot provides a powerful tool with several essential functionalities that allow the informative representation of organelle genomes. In addition to its diverse coloring schemes, Chloroplot allows a high-level interactive modification of the output, as well as the capability for plotting up to three user-provided distinct indices. The availability of the software source code provides another layer of flexibility that enables more advanced R users to further manipulate outputs as needed.

## Data Availability Statement

Publicly available datasets were analyzed in this study. This data can be found here: https://www.ncbi.nlm.nih.gov/genome/organelle/.

## Author Contributions

The study was conceived by AA and PP. Formal analysis, investigation, methodology, and software development were carried out by AA and SZ. AA carried out visualization, while PP contributed to data curation and validation. The study was supervised and financially supported by JH and JT. The original draft was written by AA and PP. All authors have read, edited, and approved the final version of the manuscript. The authors thank Jacquelin DeFaveri for assistance editing the manuscript.

### Conflict of Interest

The authors declare that the research was conducted in the absence of any commercial or financial relationships that could be construed as a potential conflict of interest.

## Footnotes

^1^https://www.darwintreeoflife.org/

## Supplementary Material

Supplementary Table S1 List of chloroplast and mitochondrial genomes used to test Chloroplot.
