# Supplementary material for: Chloroplot: An Online Program for the Versatile Plotting of Organelle Genomes
Source: Front Genet. 2020 Sep 25;11:576124. doi: 10.3389/fgene.2020.576124 (PMC7545089; doi:10.3389/fgene.2020.576124)
Supplement: Supplementary file 1 [file Data_Sheet_1.pdf]

Figure S1. Comparison of genome maps generated by major organelle visualization tools. The complete chloroplast genome sequence of *Guizotia abyssinica* (NC010601) was visualized with OGDraw v1.3.1 (**A**), CPGAVAS2 (**B**), GenomeVx (**C**) and Chloroplot (**D**) using default settings of each program. OGDraw and CPGAVAS2 assumes that the sequences of the inverted repeats (IR) are identical. Searches in each case are terminated when mismatches are identified. Both OGDraw and CPGAVAS2 recognized the "AA" indel event of a poly-A repeat in the IRs of *G. abyssinica* found in the *trn*I-GAU gene (133,365 – 133,366 bp). The default options terminated the search for IRs at the position of a T/C (104,926/130,354 bp) in the rrn23 gene. The location of this T/C mismatch serve as ultimate bases for the IRs depicted in Figure S1A and S1B. For detailed description see the publication of each software. Here we highlight differences found among the default options of each program.

Figure S1A. The genome map of *G. abyssinica* plotted with default settings of OGDraw v1.3.1 (Lohse et al. 2007, 2013; Greiner et al. 2019). Error detection should be carried out manually, non-identical IRs resulting from assembly or sequencing errors can be visualized using custom settings (figure not shown). Genomes can be plotted in circular or linear forms. Annotation can be carried out with GeSeq (Tillich et al. 2017) available from the Chlorobox website. The program also allows plotting mitochondrial genome maps.

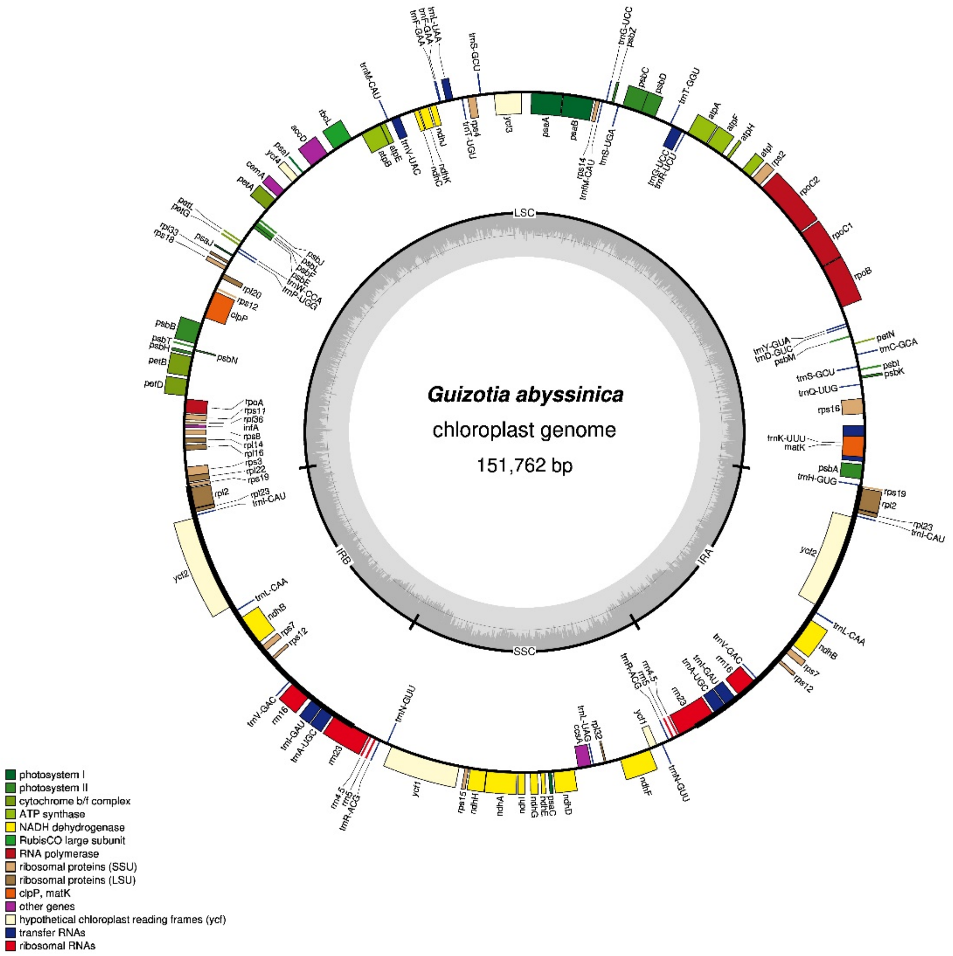

Figure S1B. The genome map of *G. abyssinica* visualized with CPGAVAS2 (Liu et al. 2012; Shi et al. 2019). The end points of the IRs are referring to the position of the T/C (104,926/130,354 bp) mismatch found in the rrn23 as shown in our figure. CPGAVAS2 lacks custom settings for IR visualization thus non-identical sequences can be plotted by manually correcting the errors in the FASTA file. CPGAVAS2 plots forward and reverse repeats and microsatellites in the chloroplast genome sequence. Annotation is integrated with genome visualization. Mitochondrial genome maps cannot be plotted.

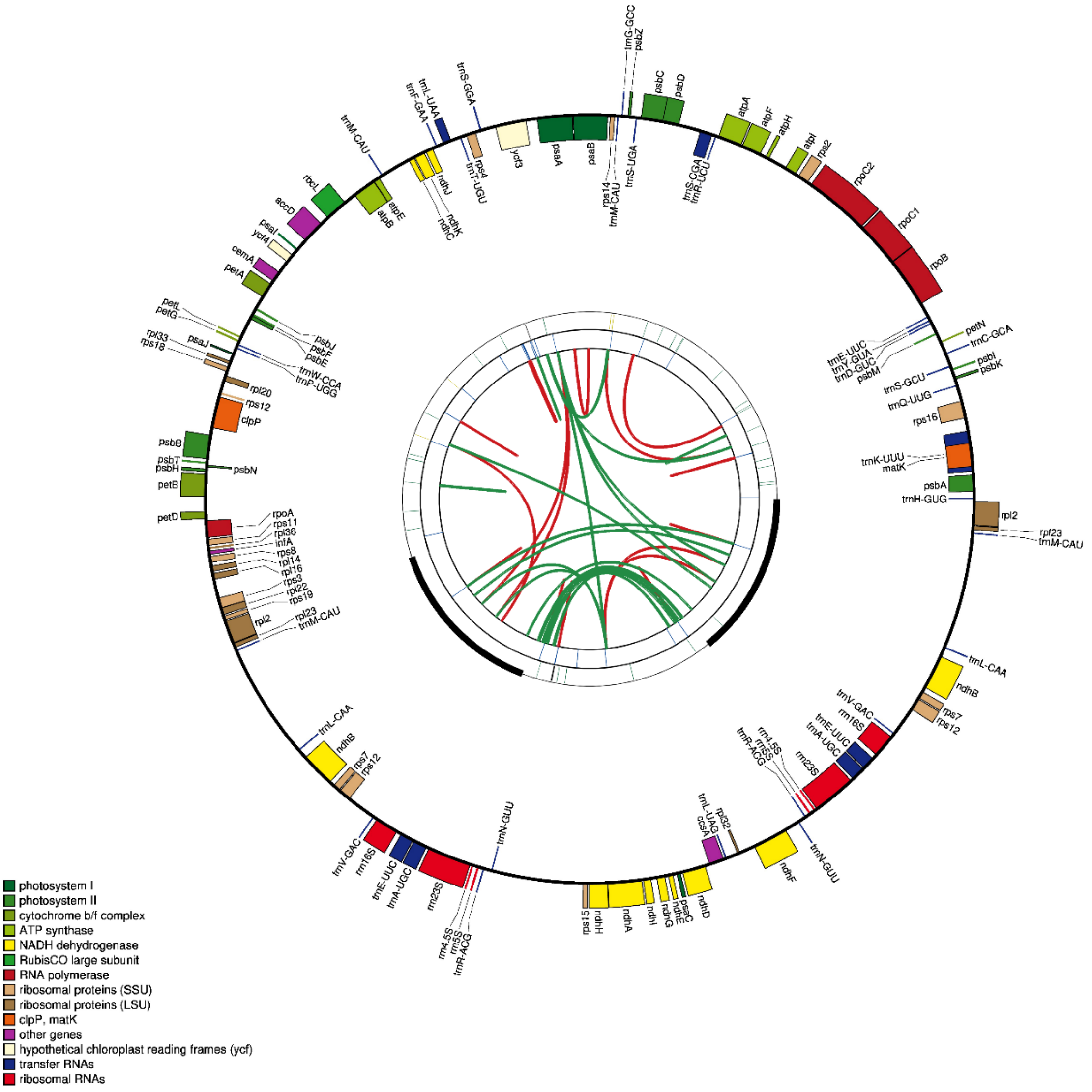

Figure S1C. The genome map of *G. abyssinica* visualized with GenomeVx (Conant and Wolfe 2008). The software offers different coloring option, while the co-ordinates of the IR regions should be given manually. The program relies on annotated GenBank files or requires the manual input of gene locations. Various genome sequences can be plotted in circular format including chloroplast and mitochondrial genomes as well as plasmids.

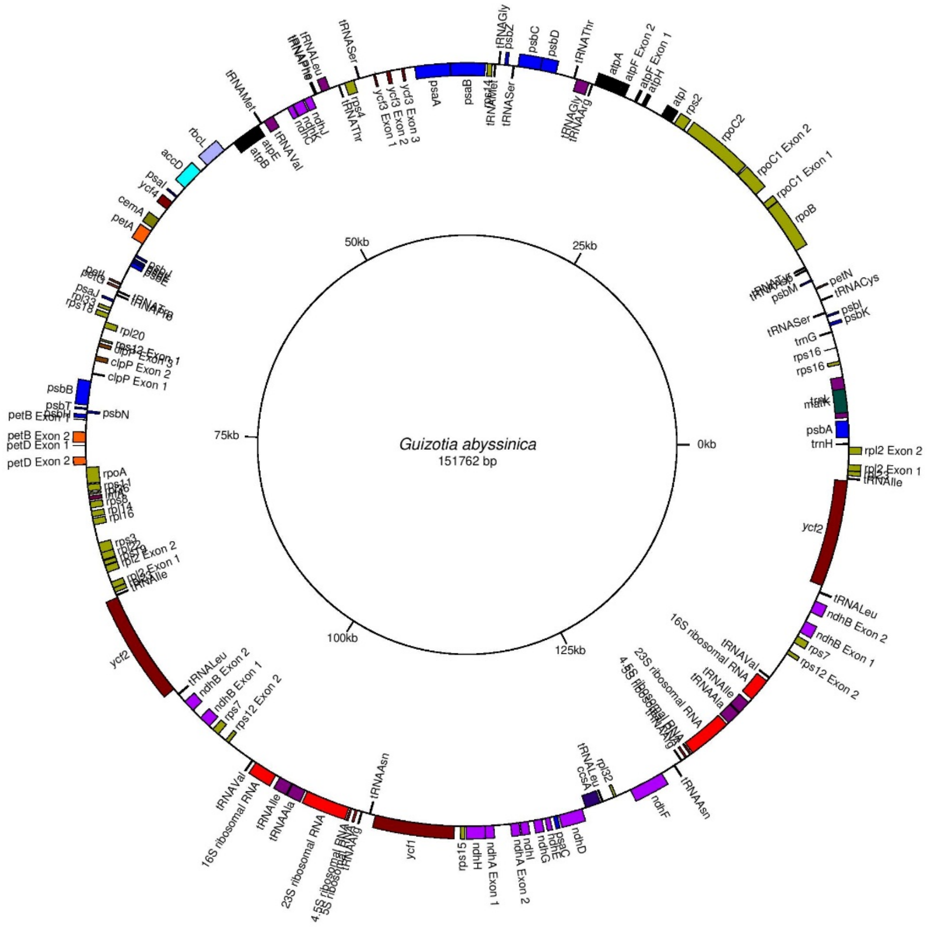

Figure S1D. The genome map of *G. abyssinica* visualized with Chloroplot. The program allows the presentation of non-identical IRs with default settings. The co-ordinates of the inverted repeats are plotted based on a custom sequence search with a minimal threshold of 100 bp implemented in the software. In case of *G. abyssinica*, the non-identical IRs are highlighted with blue. Differences in the length (IRB: 25,001 bp) are caused by the 2-bp "AA" deletion detected in the rrn23 gene (IRA: 24,999 bp). Insertions and deletion are highlighted with green and yellow dots. Red dots mark several point mutations (SNPs) among the two IR copies, possibly arising from sequencing and/or assembly error.

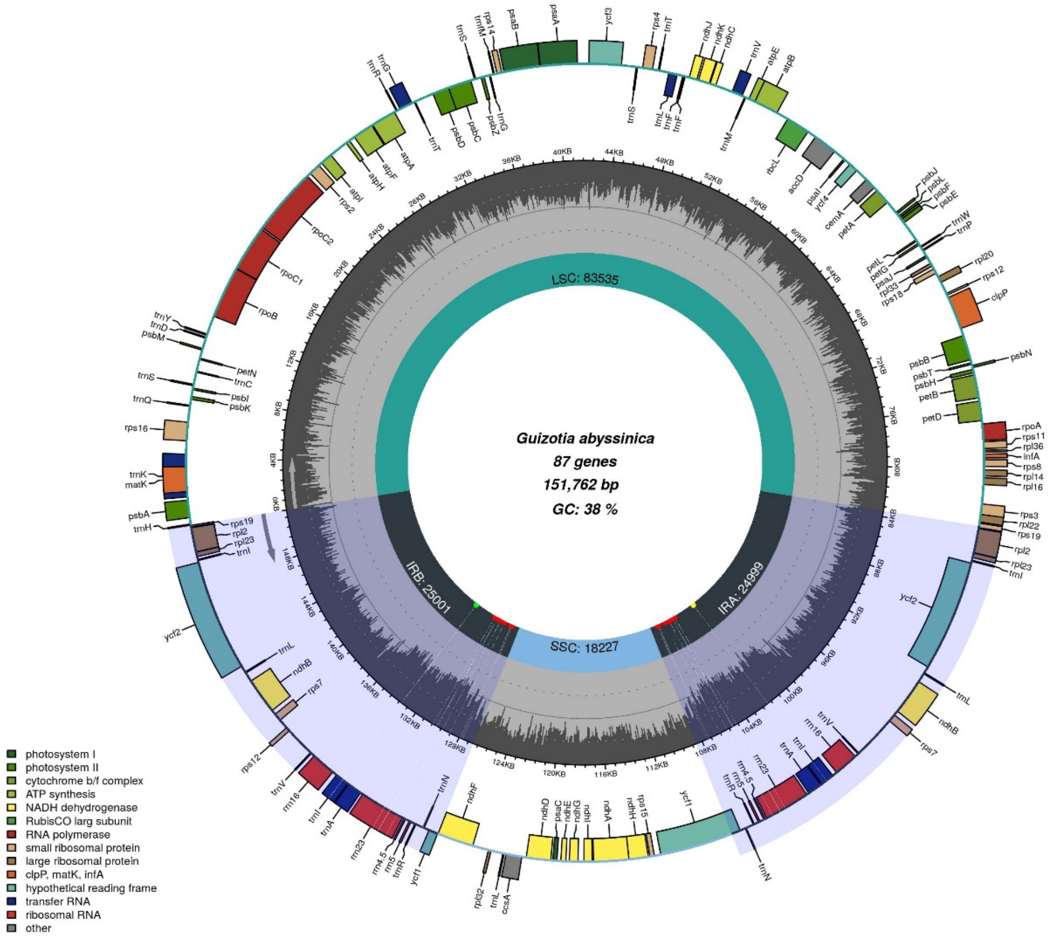

References

Conant, G.C. and Wolfe, K.H. (2008) GenomeVx: simple web-based creation of editable circular chromosome maps. Bioinformatics 24, 861–862.

Greiner, S., Lehwark, P. and Bock, R. (2019) OrganellarGenomeDRAW (OGDRAW) version 1.3.1: expanded toolkit for the graphical visualization of organellar genomes. Nucleic Acids Res. 47, W59–W64.

Liu, C., Shi, L., Zhu, Y., Chen, H., Zhang, J., Lin, X. and Guan, X. (2012) CpGAVAS, an integrated web server for the annotation, visualization, analysis, and GenBank submission of completely sequenced chloroplast genome sequences. BMC Genomics 12, 715.

Lohse, M., Drechsel O., and Bock, R. (2007) OrganellarGenomeDRAW (OGDRAW): a tool for the easy generation of high-quality custom graphical maps of plastid and mitochondrial genomes. Curr. Genet. 52, 267–274.

Lohse, M., Drechsel, O., Kahlau, S. and Bock, R. (2013) OrganellarGenomeDRAW – a suite of tools for generatic physical maps of plastid and mitochondrial genomes and visualizing expression data sets. Nucleic Acids Res. 41, W575–W581.

Shi, L., Chen, H., Jiang, M., Wang, L., Wu, X. and Huang, L. et al. (2019) CPGAVAS2, an integrated plastome sequence annotator and analyzer. Nucleic Acids Res. 47, W65–W73.

Tillich, M., Lehwark, P., Pellizzer, T., Ulbricht-Jones, E.S., Fischer, A. and Bock, R. et al. (2017) GeSeq – versatile and accurate annotation of organelle genomes. Nucleic Acids Res. 45, W6–W11.
